# COX‐2 as a potential blood based biomarker and a possible therapeutic target in Alzheimer’s Disease

**DOI:** 10.1002/alz.090807

**Published:** 2025-01-09

**Authors:** Sakshi Kumari, Sharmistha Dey

**Affiliations:** ^1^ All India Institute of Medical Sciences, New Delhi India; ^2^ All India Institute of Medical Sciences, AIIMS, New Delhi, Delhi India

## Abstract

**Background:**

Alzheimer’s disease (AD) is a progressive brain disorder which leads to gradual decline in memory, thinking, behaviour and social skills. The current scenario for drug development is based on neuro‐inflammation and oxidative stress. Amyloid‐β (Aβ) deposition, a major hallmark of the disease activates microglia leading to neuro‐inflammation and neuro‐degeneration induced by activation of COX‐2 via NFkB p50 in glioblastoma cells.

**Method:**

The level of proteins and their mRNA in blood of study groups were measured by surface plasmon resonance(SPR) and further validated by western blot and quantitative polymerase chain reaction (qPCR), respectively. The binding of designed peptide with COX‐2 was confirmed by SPR. Also, the rescue of neurotoxicity by peptide was checked by MTT assay on SH‐SY5Y cells (neuroblastoma cell line).

**Result:**

Proteins and mRNA were found to be highly expressed in AD and MCI compared to GC. However, level of COX‐2 decreases with disease duration. The peptide showed binding affinity with COX‐2 with low dissociation constant in SPR and rescued the neurotoxicity of SH‐SY5Y cells by decreasing the level of Aβ, Tau and pTau proteins.

**Conclusion:**

It can be concluded that COX‐2 protein can serve as a potential blood‐based biomarker for early detection and can be a good platform for therapeutic intervention for AD.

